# Editorial

**Published:** 1900-05-15

**Authors:** 


### EDITORIAL.

FOR the first time in several years an effort is being made to hold clinics in Operative Dentistry at the meeting of the National Dental Association, at Old Point Comfort, Va., in July. It is to be hoped that all who can will contribute to make the venture a successful one. The section has promised to provide facilities, and it is hoped to make it one of the valuable features of the meeting.

ON the evening of April 7th a considerable number of the profession in the East gathered in New York City to attend a banquet given in honor of Dr. Norman W. Kingsley. The occasion was made ostensibly to celebrate his fiftieth anniversary as a practitioner of dentistry. From the account printed in the May *Items of Interest*, we conclude that it was an entirely satisfactory review of his professional career. His record shows that, while he has made the profession of dentistry his chief work, he has also cultivated other attributes of his nature and character. By his work as a sculptor he has made fame for himself as an artist. Our St. Louis brethren have also banquetted that prince of men and everywhere well-known dentist, Dr. H. J. McKellops, of St. Louis. He too, like Dr. Kingsley, has shown a capacity for art, and cultivated in his early days literature, especially the drama. His reading of the poem, "A Cup of Cold Water," shows his artistic nature and culture. It is well to honor such men and take such means as may be practicable, not only to bestow these honors while they can be received and enjoyed by their recipients, surrounded by sympathetic friends, and at a time in life when they will bring the greatest pleasure and satisfaction; but they have also another important function that is none the less valuable, they serve to give the younger men an insight into the fields of higher opportunity, and to stir up ambition to spur them on to "make their lives sublime," as the poet puts it. It is right and proper for us to honor our dead, but how much greater privilege it is to honor our living. Let us have more banquets, if they are needed to furnish us opportunity to show our appreciation of the noble, self-sacrificing lives which are being lived in our very midst, it may be.

It has long been a mystery to us how the *Indiana Dental Journal* could put up one hundred pages of as good reading as it does each month for a dollar. But we think that editorial in this month's issue on "The Fable of the Sporty Dentist" gives us a clue, at least. We supposed Brother Hunt did all the writing himself, but we can never believe his social life was such as acquaint him with the slang vernacular used; nor would his former and excellent writing account for the intolerable abuse of the place of the capital letter; and lastly, such a narrative could surely be generated only by continued indulgence in literature, such as boys with a burning desire to be "*tough*" are inclined to spend their time. It must have been the office boy.

